# Ultrastructural changes during osteogenic differentiation in mesenchymal stromal cells cultured in alginate hydrogel

**DOI:** 10.1186/s13578-016-0128-0

**Published:** 2017-01-03

**Authors:** Jakub Grzesiak, Agnieszka Śmieszek, Krzysztof Marycz

**Affiliations:** 1 Electron Microscopy Laboratory, Wroclaw Research Centre EIT+, Stabłowicka 147, 54-066 Wrocław, Poland; 2 Electron Microscopy Laboratory, Wroclaw University of Environmental and Life Sciences, Kożuchowska 5b, 51-631 Wrocław, Poland

**Keywords:** Mesenchymal stromal cells, Alginate hydrogel, Osteogenic differentiation, Mineralizing vesicles, Apoptosis, Ultrastructure

## Abstract

**Background:**

Osteogenic differentiation of mesenchymal stem cells has been extensively investigated with regards to different aspects, including the analysis of cell intracellular and extracellular proteome, cell gene expression pattern, and morphology. During the osteogenic differentiation, osteoblasts produce and release specific proteins, like osteocalcin and osteopontin. Simultaneously, cells produce the extracellular matrix (ECM) that resembles the bone ECM, with high quantity of calcium and phosphorus. We focused on the ultrastructural changes occurring during the osteogenic differentiation of MSC cultured in alginate hydrogel.

**Results:**

The analysis revealed that during the osteogenic differentiation the most of cells become dead, and these dead cells contain large quantities of calcium and deposition is strictly connected with the cellular death and small membrane vesicles released by cells. Cell organelles were not present within differentiated cells, while in cells from non-osteogenic group the cellular ultrastructure was proper, with single nuclei, endoplasmic reticulum and numerous mitochondria.

**Conclusion:**

The ECM synthesis and deposition during the osteogenic differentiation of MSC involves cellular programmed death. The small membrane vesicles become the mineralization sites of formed bone ECM.

**Electronic supplementary material:**

The online version of this article (doi:10.1186/s13578-016-0128-0) contains supplementary material, which is available to authorized users.

## Background

Osteogenic differentiation in vitro by mesenchymal stromal cells (MSC) is the process involving the change in cell behavior, protein secretion profile and cell ultrastructure. While this process is well analyzed in monolayer cell cultures [1], the osteogenic differentiation in 3D hydrogel culture model needs to be enclosed, especially concerning the ultrastructural features of cells undergoing the differentiation, and the mechanisms of ECM deposition.

Mesenchymal stem cells (MSC), which have been extensively used in experiments and clinical trials are characterized by their multilineage differentiation potential, which means that they are able to differentiate into the specialized cells, like osteoblasts, adipoblasts or chondroblasts, among others [2]. During the osteogenic differentiation, MSC change their behavior, morphology, gene and proteome expression patterns. For example, in MSC cultured in osteogenic conditions the alkaline phosphatase becomes active, which is followed by the synthesis and secretion of osteocalcin protein. Simultaneously, the process of biomineralization occurs [3]. Nonetheless, the exact mechanism of matrix synthesis and calcification is still not clear enough. Anderson [4] showed with electron microscopic techniques that during the biomineralization occurring in the cartilage, bone and dentin tissues, the abundant small vesicles—called matrix vesicles (MV), could be found at the initial calcification sites [4].

Matrix vesicles, also known as microvesicles are small extracellular organelles which arise for example by budding from cell surface, therefore they are composed of small cytoplasm fragment surrounded by the double layered plasma membrane. They have been identified in all tissues, but their role is still not sufficiently explained. They constitute the one of intercellular communication mechanisms, as it was shown in previous studies [5]. Moreover, numerous observations of Anderson [4, 6–12] and others revealed that similar submicron organelles may be also involved in the process of biomineralization [4, 6–12].

In 2009 Golub described the three hypothetical mechanisms on which the calcification of extracellular matrix might be based [13]. One of the hypothesis assumes, that MV released by osteoblasts only regulate the concentrations of ions outside the MV, which leads to the formation of soluble molecular species, followed by the initiation of mineral formation in collagen. Second hypothesis states that the apatite crystals are formed within the MV, which is followed by their release and deposition over the collagen fibers. Third hypothesis assumes that besides the accumulation of calcium phosphate within the MV, its further propagation is facilitated by the interaction between MV and collagen fibrils. These hypotheses has not been proven nor excluded yet. Moreover, it was noticed by Proudfoot et al. that during the osteogenic differentiation, the osteoblasts loss their viability and undergo apoptosis, and the apoptotic bodies establish the nucleation sites for newly formed mineralized matrix [14].

In this work we analyzed if the process of osteogenic differentiation of MSC cultured in calcium alginate beads occurs also by means of similar mechanisms to in vivo conditions, with the major attention put on ultrastructure of cells and the process of biomineralization at the nano-scale level—the features that have not been analyzed at this scale yet. We showed that indeed the microvesicles may become the mineralization sites for forming hydroxyapatite crystals. Moreover, we showed that the ultimately differentiated cell become dead and filled with hydroxyapatite.

## Methods

### Preparation of alginate solution

Sodium alginate solution was prepared from low viscosity sodium alginate (Sigma Aldrich) dissolved in sterile 0.9% NaCl at a final concentration of 2% w/v. Obtained solution was sterile filtered using 0.45 and 0.22 µm syringe filters and stored in 4 °C for further usage.

### Cell culture

Bone marrow mesenchymal stem cells (BMSC) were isolated from adult rat of Wistar bred, using method described before [15]. Briefly, the femurs and tibias were collected directly after the euthanasia of the donor and placed in sterile Hank’s balanced salt solution (HBSS). For cell collection, bone heads were cut and the marrow was flushed out from the bone with Dulbecco modified eagle medium (DMEM) to a falcon tube, followed by centrifugation (300×*g*/4 min) and resuspension in DMEM containing the 10% of fetal bovine serum (FBS) and 1% of penicillin/streptomycin (standard medium). Cells were plated in T-75 culture flasks for primary culture. Cells were propagated for 5–7 days until they reach full confluence.

At this stage of culture, cells positive for specific antigens of mesenchymal stem cell population, i.e. CD44^+^/CD90^+^ were isolated, and the remaining cells discarded, including cells positive for hematopoietic cell marker CD45^+^ by means of magnetic, immuno-based separation technique (rabbit anti-rat CD44, 3 µg/ml, Sigma Aldrich; mouse anti-rat CD90, 5 µg/ml, Sigma Aldrich; anti-rabbit/anti-mouse IgG-magnetic microbeads labeled, MACS, Miltenyi Biotec, Germany). Purified BMSC were centrifuged 300×*g*/4 min and resuspended in 2% sodium alginate solution at a concentration of 5 × 10^5^ cells/ml as the secondary cultures. To obtain calcium alginate gels with BMSC embedded within, the 50 µl-droplets of cell suspension in alginate solution were dropped in 0.1 M CaCl_2_ and kept in for 5 min at room temperature. Materials were washed three times in DMEM and placed in 96-well plate (every hydrogel sample in separate well) for osteogenic differentiation assay. Prior to assay, two samples were taken for evaluation of cell viability, using calcein AM and propidium iodide fluorescent staining and microscope, which resulted in over 95% of cell viability. Cultures were maintained in osteogenic differentiation medium (StemPro Osteogenesis Differentiation Kit, Life Technologies), and in standard medium (DMEM with l-glutamine, 10% of fetal bovine serum). Cell culture media were changed twice a week, cell differentiation was maintained for 3 weeks.

### Microscopic observations

After the three weeks of culture, hydrogel samples were stained for viability using calcein AM and propidium iodide, using standard protocol. Other samples were fixed in 2.5% glutaraldehyde overnight in 4 °C for electron microscopic analysis, in 4% neutral buffered formalin for 1 h for H&E and histochemistry, and in 4% paraformaldehyde for immunofluorescence. Prior to histological staining, hydrogels were dehydrated in graded series of ethanol, embedded in paraffin, cut on 5 µm sections and collected on microscopic slides. Sections were stained with hematoxylin (3 min) and eosin (2 min), washed with water and covered with coverslips using DPX. Preparations were analyzed with light microscope (AxioImager, Zeiss). Documentation was made with Canon PowerShot digital camera.

To analyze the presence of apoptotic processes during the differentiation, immunofluorescent staining for caspase-3 protein was performed. Paraformaldehyde-fixed hydrogels were washed three times with PBS containing 2% of FBS. Next, samples were incubated in 0.05% Triton x-100 in PBS for 15 min, followed by triple washing and blocking in 5% normal goat serum in PBS for 1 h. After blocking, samples were washed three times in PBS and incubated overnight with primary antibody (mouse anti-casp3, Thermo Scientific, cat. no. 437,800, concentration 1:100). Next, samples were washed three times with PBS and incubated with secondary antibody solution (goat anti-mouse IgG conjugated to Atto-629, Thermo Scientific, concentration 1:500) for 1 h at 37 °C. After triple washing in PBS, samples were counterstained with 4′,6-diamidino-2-phenylindole (DAPI, 5 µg/ml) and observed in confocal microscope as a whole mount (Zeiss Axio LiveImager). To prepare a simple 3D reconstruction of the signals, the z-stacked images were processed using Imaris software.

For electron microscopic observations, samples were prepared as critical point-dried (CPD) fractures of whole hydrogels with cells, and as heavy metal-stained (HM) samples embedded in resin. Prior to observations, CPD samples were undertaken for conduction-providing preparation (OTOTO protocol) [16]. Briefly, fixed samples were washed in cacodylate buffer (pH = 6.8) for glutaraldehyde removal and incubated in cacodylate-buffered 1% osmium tetroxide with 1.5% of potassium ferricyanide for 1 h. After washing three times in buffer and two times in ultrapure water (UPW), samples were incubated in 1% thiocarbohydrazide (TCH) aqueous solution for 30 min. Next, samples were washed 5 times in UPW and incubated in 2% osmium tetroxide in cacodylate buffer for 30 min, followed by another five washings in UPW and incubation in TCH solution for 30 min. After another five washings in UPW, samples were incubated in 2% osmium tetroxide in cacodylate buffer for 20 min, washed 5 times in UPW and dehydrated using graded series of acetone (from 10% up to 100% of acetone concentration, change every 10%). At this stage, hydrogels were sectioned using surgical blade, and dried using automatic critical point dryer (CPD300, Leica). Dried samples were mounted on microscope stub and placed in scanning electron microscope (Auriga 60, Zeiss) for observations and elemental composition analysis. Observations were made at 2 kV of filament tension. The focused ion beam milling was performed to reveal the internal structure of cells (FIB, Cobra, Auriga 60, Zeiss).

HM samples were fixed in 2.5% glutaraldehyde in cacodylate buffer (pH = 6.8) for 24 h, followed by their triple washing in buffer and incubation in buffered 1% osmium tetroxide with 1.5% of potassium ferricyanide for 1 h on ice. Next, samples were washed three times in buffer and three times in UPW. Material was then incubated with 1% uranyl acetate aqueous solution overnight at 4 °C. After five washings in UPW, samples were dehydrated using graded acetone series, incubated in agar resin (1:3, 1:1, 3:1 of resin:acetone and pure resin, 2 h each). Resin blocks were polymerized for 48 h at 60 °C. Samples were then trimmed with glass knife using ultramicrotome (AC7, Leica) to prepare the surface for FIB milling. Trimmed samples were mounted on microscope stub with carbon tape, covered with 20 nm layer of gold with high vacuum coater (EM ACE600, Leica), and placed in microscope chamber (Auriga 60, Zeiss). The cells were found by observations of polished surface at 20 kV of beam acceleration voltage, where the single cells were visible. Prior to FIB milling, milled surface was covered with protective 5 nm layer of platinum using gas injection system. Materials were milled using 30 kV/16 nA aperture to obtain the trench, and 30 kV/2 nA to polish the imaged surface. Cells were then observed using SE2 detector at 2 kV of acceleration voltage and LUT display set on inverted mode.

### Markers of osteogenic differentiation

#### Alizarin Red S staining

To analyze the presence of calcium ECM in hydrogels, Alizarin Red S staining was applied. Briefly, fixed hydrogels were washed in distilled water, incubated for 10 min in Alizarin Red S working solution, washed three times in water and observed with inverted microscope as a whole mount. Additionally, fixed hydrogels were embedded in paraffin, cut for 5 µm sections and stained for 5 min. Documentation was made with Canon PowerShot digital camera.

#### SEM–EDX measurements

Energy dispersive X-ray (EDX) analysis of calcium and phosphorus content within the hydrogels was performed using Oxford detector and Inca software combined with SEM at 20 kV of accelerating voltage. For this analysis, critical point-dried, fractured samples were taken. Three repeats from every group were made, and the results were averaged and statistically verified using Student’s *t* test. The distribution of calcium and phosphorus was determined at the FIB-milled area to show the concentration of these elements around the single cells.

#### Measurements of alkaline phosphatase, osteocalcin and osteopontin

Extracellular activity of ALP was determined in the supernatants collected during the last day of experimental culture. The assay was performed using the alkaline phosphatase colorimetric assay kit (Abcam, Cambridge, UK), according to protocol provided by the manufacturer. The experimental samples were prepared in duplicates and were diluted twofold. Sample background control was included and the background was corrected by subtracting the value derived from the zero standards from all standards, samples, and sample background control. As a phosphatase substrate, the p-nitrophenyl phosphate (pNPP) was used. The substrate was hydrolyzed into p-nitrophenol by alkaline phosphatase. The reaction product was measured at 405 nm of wavelength using microplate reader (BMG Labtech). Sample readings were applied to the standard curve to obtain the amount of pNP generated by the ALP sample. The enzymatic activity was determined using the following formula:

ALP activity (U/ml) = A/V/T, where: (i) A is the amount of pNP generated by the samples (in nmol); (ii) V is the volume of sample added to the assay well (in ml) and (iii) T is the reaction time.

The osteocalcin (OCL) and osteopontin (OPN) levels were determined in culture supernatants collected after the last day of culture, from three plate wells (repeats) for each analyzed group. Analyzed protein content was determined using Rat Gla-Osteocalcin High Sensitive ELISA Kit (Takara) and Mouse/Rat Osteopontin Quantikine ELISA Kit (R&D Systems). The quantitative determination of OCL and OPN was performed according to manufacturers’ instructions. The amount of detected proteins was expressed as protein weight/supernatant volume ratio (w/v). Statistical significance of obtained results was verified using OneWay Anova test.

## Results

### Microscopic observations

**Fig. 1:**
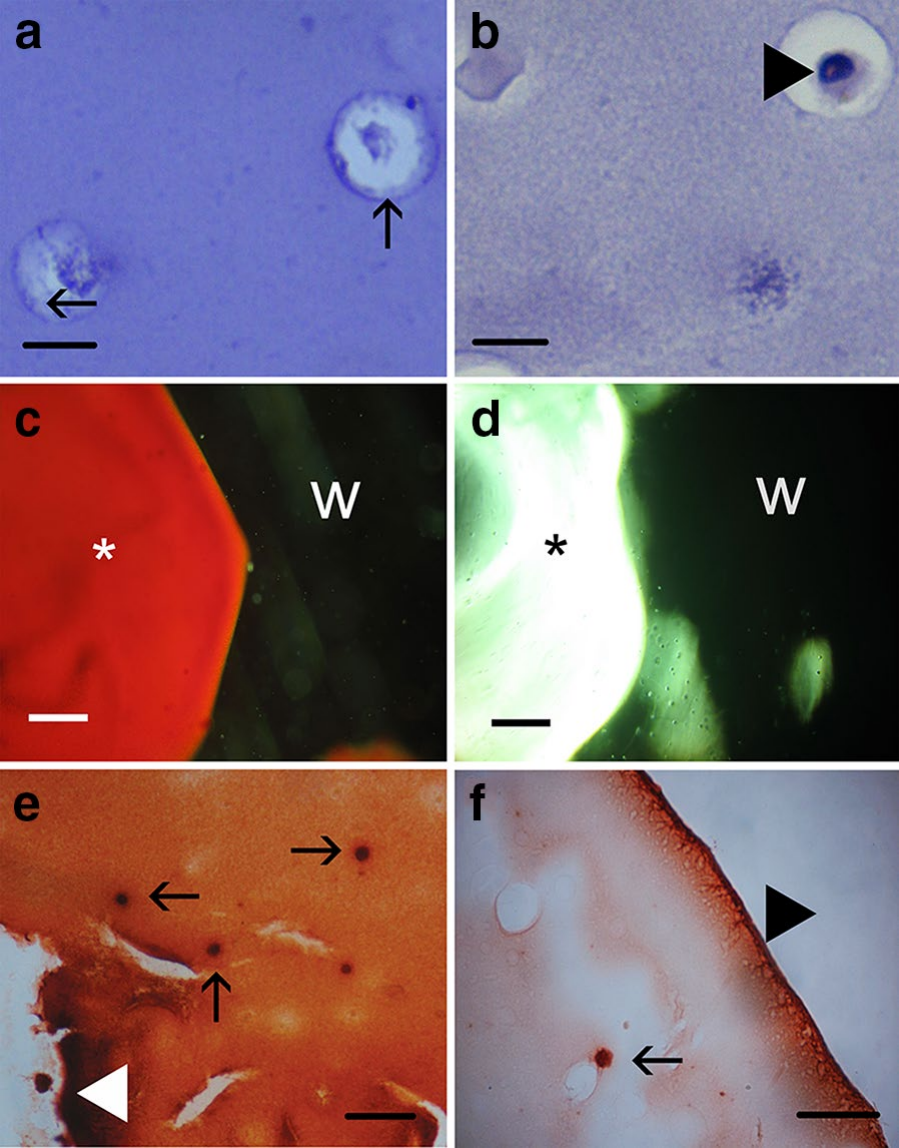
The histological differences between hydrogels from osteogenic and non-osteogenic culture conditions. H&E staining revealed differences in cell appearance between groups (**a** osteogenic, **b** non-osteogenic, *black arrows* show blurred cavity border, *black arrowhead* show the nucleus, *scale bars* 20 µm). Alizarin Red S staining indicated the high calcium ion content evenly distributed within the hydrogel in osteogenic group (**c**), whereas in control hydrogel was not stained (**d** *hydrogel, *w* culture well, *scale bars* 400 µm). The analysis of histological sections showed strong Alizarin Red S reaction of cells (*black arrows*) and hydrogel’s edges (*white arrowheads*) in osteogenic group (**e**), while in control only single cells (*black arrow*) and the superficial zone of hydrogel (*black arrowhead*) were positively stained (**f** *scale bars* 200 µm)

In samples maintained in osteogenic medium, observations showed increased turbidity of hydrogel occurred during three-week culture. The borders of materials were prominently detectable, with visible wavy shape and conspicuous cracks present on the surface. Hematoxylin and eosin staining revealed differences in cellular morphology between groups. In osteogenic group nuclei of cells present in alginate cavities were not visible, and the inner borders of cavities were blurred (Fig. 1a). In non-osteogenic group cells had prominent, single nuclei and the borders of cavities were sharp (Fig. 1b). Alizarin Red S staining of whole hydrogels showed high concentration of calcium ions, dispersed evenly in the material from osteogenic group (Fig. 1c). The strong positive reaction was co-localized with cells. In addition, observations revealed irregular shape of hydrogels edges with strongly positive staining reaction (Fig. 1e). The control materials maintained in non-osteogenic medium were transparent, with blurred borders without any visible cracks. Histochemical staining showed the lack of calcium ions within the hydrogel, with only weakly positive areas on single cells and at the superficial zone of material. The edges of hydrogel were strongly positive and of regular, round shape (Fig. 1d, f).

**Fig. 2:**
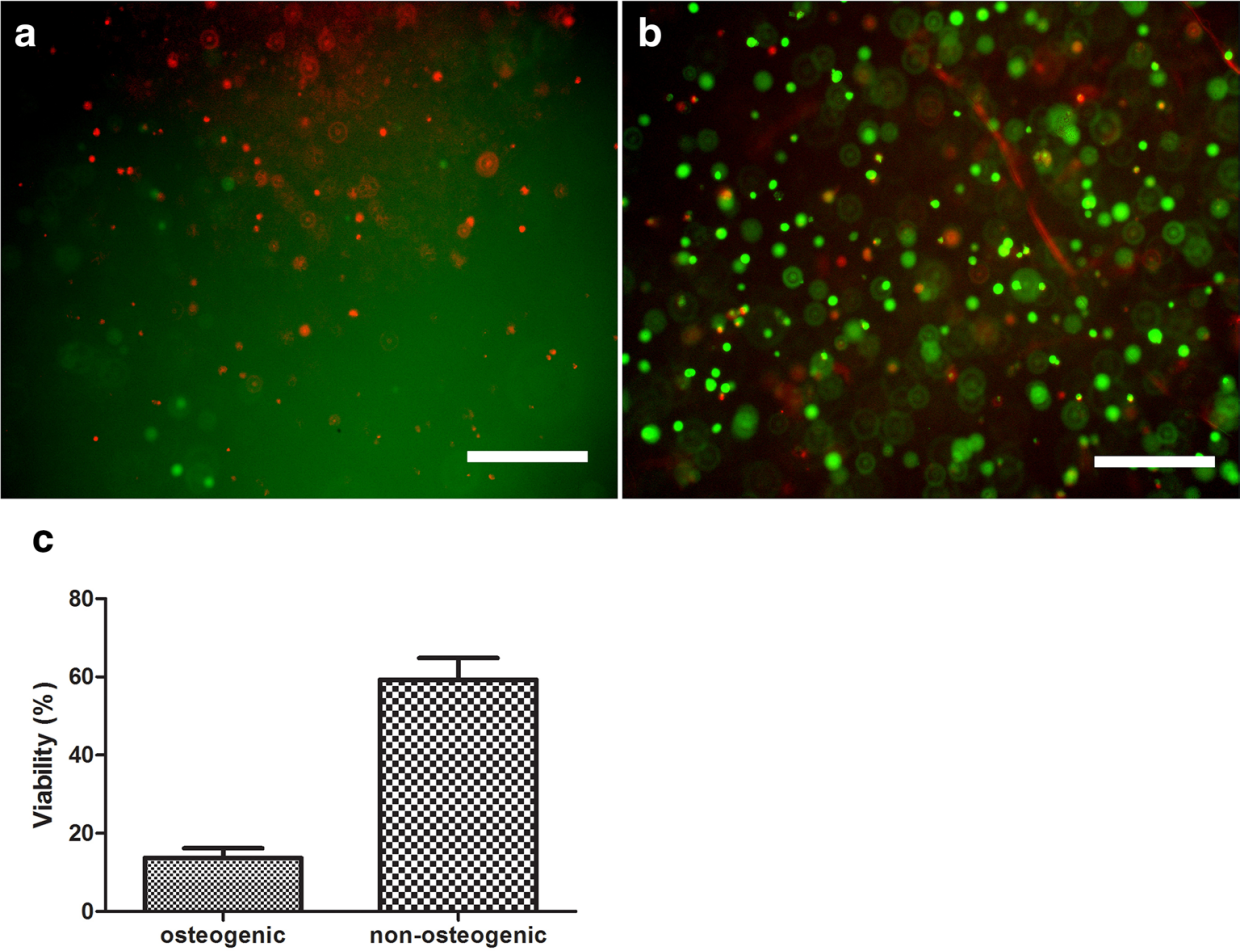
Results from live/dead staining using calcein AM (*green*) and propidium iodide (*red*) in osteogenic (**a**) and non-osteogenic (**b**) groups (images merged); the *chart* shows the percentage of viable cells within each group (**c**); *scale bar* 400 µm

**Fig. 3:**
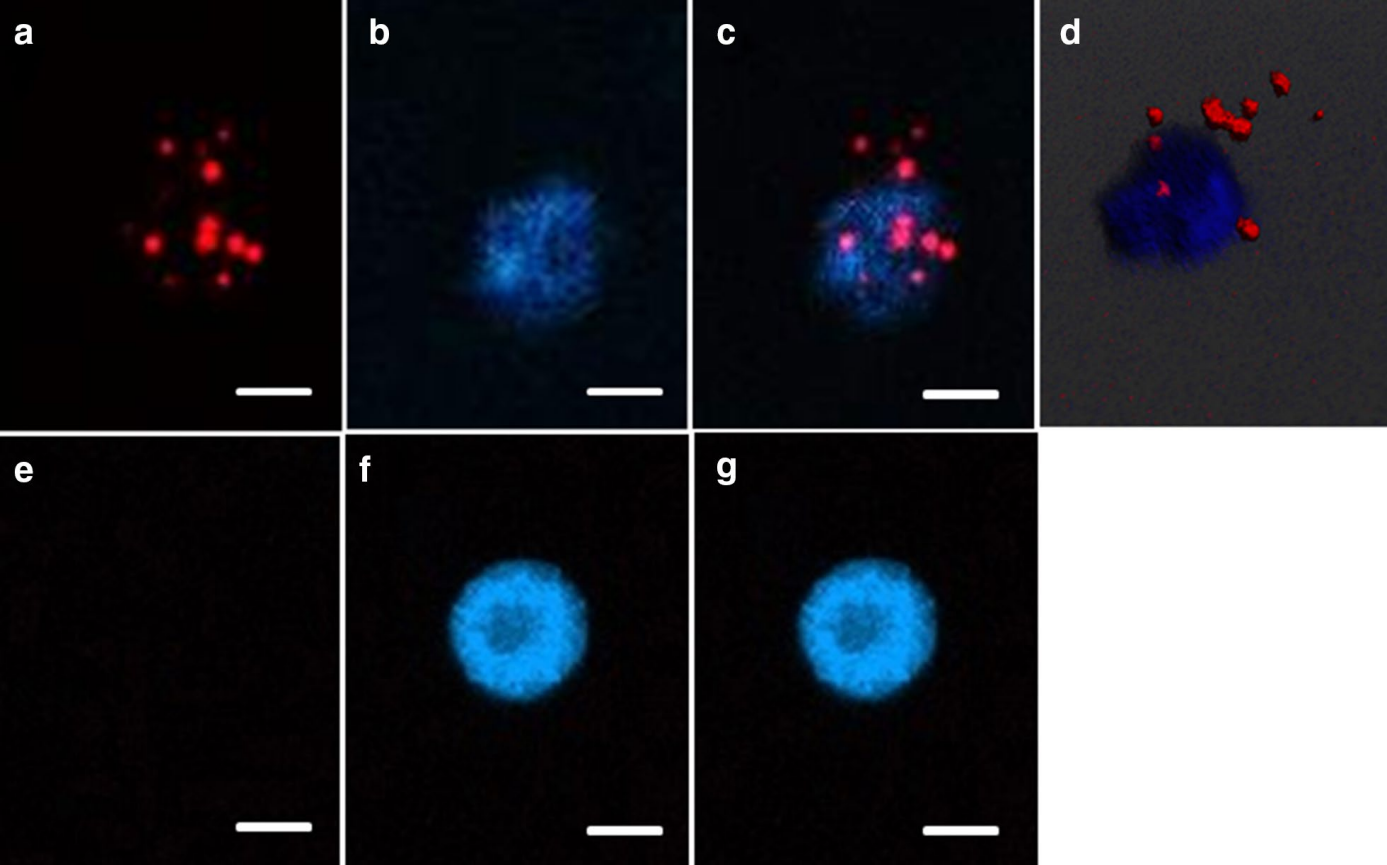
The presence of caspase-3 (*red*) indicating the apoptotic process in cell from osteogenic group (**a**–**c**), and the 3D-reconstruction of the microscopic data showing the extranuclear localization of caspase-3 signals (**d**); in cell from non-osteogenic group (**e**–**g**) the caspase-3 signal was absent; nuclei stained with DAPI (**a**, **e** caspase 3; **b**, **f** nuclei; **c**, **g** merged images; **d** 3D-reconstruction); *scale bar* 10 µm

Viability assay revealed, that in osteogenic group the majority of cells was dead after 21 days of culture. In the control group, the most of cells were viable in the hydrogel (Fig. 2). The immunofluorescent staining of caspase-3 revealed its presence in cells from osteogenic group, whereas in cells from non-osteogenic group it was not detected. The 3D-reconstruction of z-stacked images revealed that the signal was present outside the nucleus (Fig. 3) (Additional file 1).

**Fig. 4:**
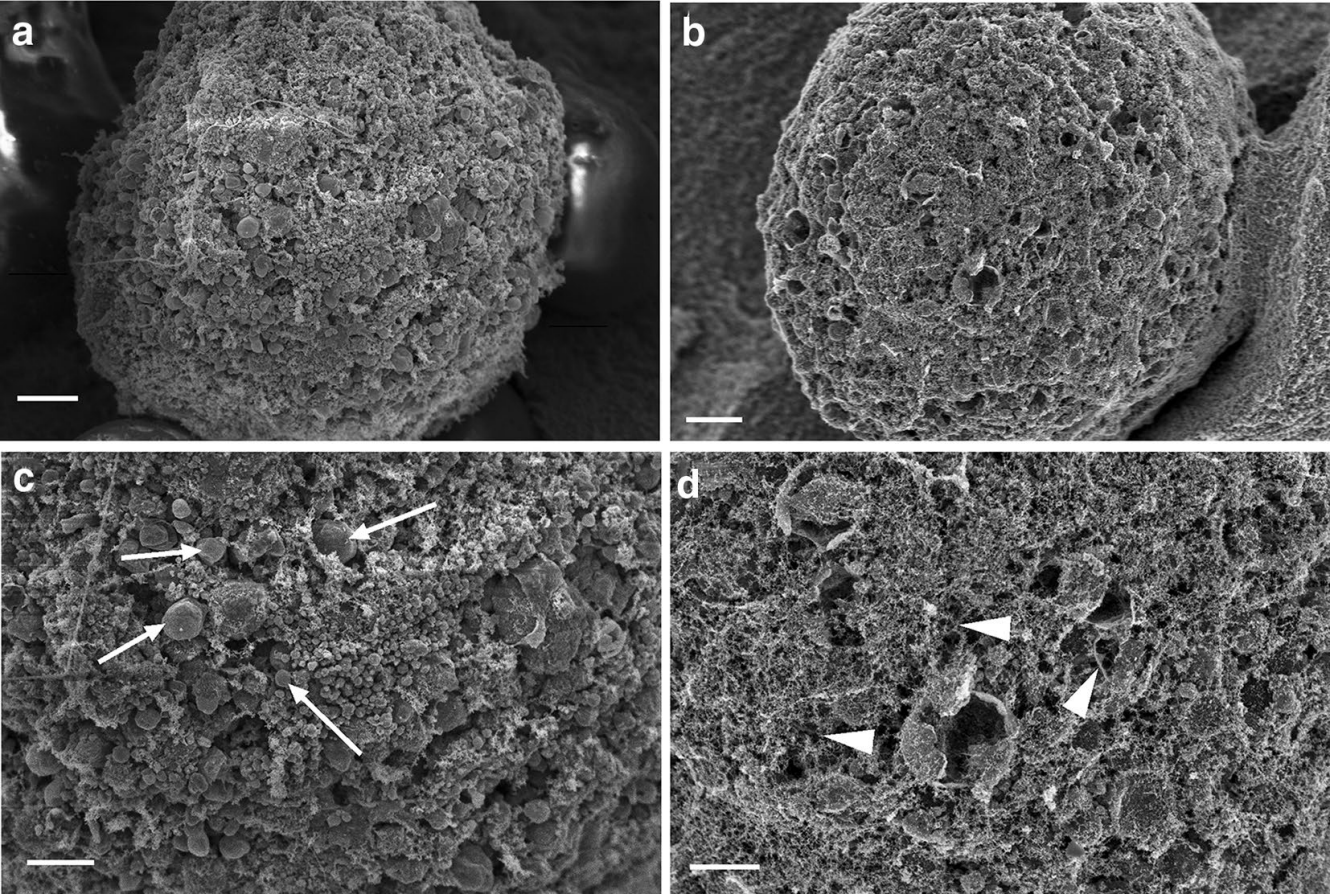
The micrographs showing the surface differences between BMSC cultured in hydrogel from osteogenic conditions (**a**, **c**, *arrows*—vesicular structures) and from non-osteogenic control (**b**, **d**, *arrowheads*—nano-sized fibers); *scale bars* 5 µm (**a**, **b**), 1 µm (**c**, **d**)

Scanning electron microscopy observations revealed differences in morphology and ultrastructure of cells from both groups. In experimental samples which were maintained under osteogenic conditions, the cell surface showed high quantity of round, vesicular-like objects, with low quantity of fibers (Fig. 4a, c). The surface of cells from non-osteogenic culture conditions was covered with fine nano-sized fibers, with low number of vesicular structures (Fig. 4b, d).

**Fig. 5:**
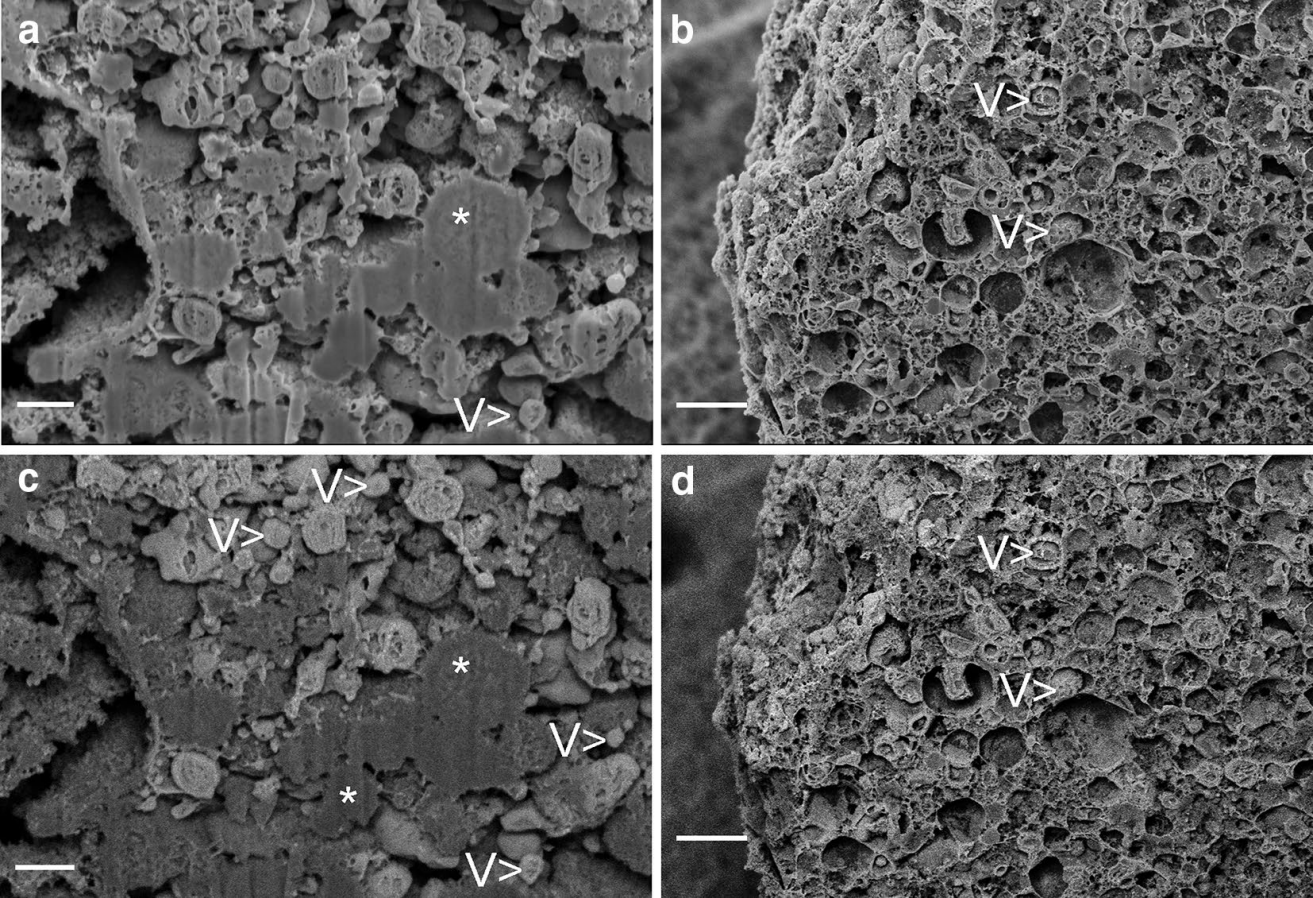
Differences in ultrastructure between BMSC cultured in osteogenic medium (**a**, **c**) and in standard medium (**b**, **d**) revealed by FIB milling and SEM observations of critical point-dried cells, captured with InLens (**a**, **b**) and ESB (**c**, **d**) detectors. *Asterisks* indicate areas composed of heavier elements, *V* vesicular structure; *scale bars* 400 nm

Observations of cell ultrastructure revealed by FIB-milling showed heterogenic character of internal components, with prominent vesicles of solid interior in osteogenic group (Fig. 5a). Energy-selective backscattered electron detector (ESB) revealed that these solid areas were composed of heavier elements than cellular structures surrounding them (Fig. 5c). Cells from non-osteogenic group were characterized by mostly vesicular internal structure, however these vesicles were rather empty (Fig. 5b), and only small fragments of cell interior were composed of heavier elements as it was noticed by means of ESB detector (Fig. 5d).

**Fig. 6:**
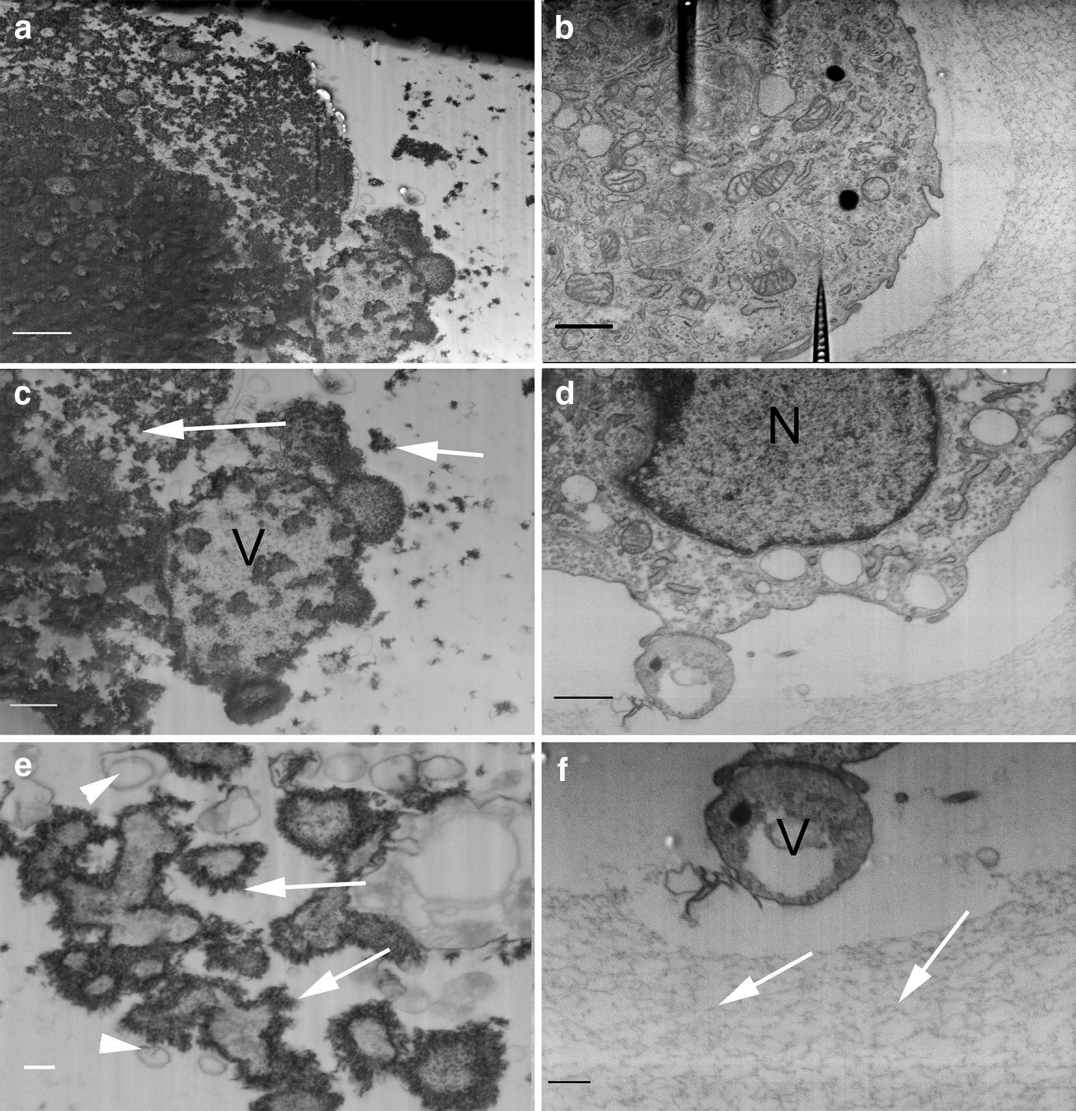
Differences in the ultrastructure between BMSC cultured in osteogenic (**a**, **c**, **e**) and non-osteogenic (**b**, **d**, **f**) conditions, revealed by FIB milling and observed with SEM as block-face low voltage imaging. In osteogenic group the organelles were not visible, and whole cell interior was filled with electron-heavy hydroxyapatite crystals (**a**), the vesicular structures were filled with hydroxyapatite deposits (**c**), and small vesicles with hydroxyapatite crystals on their membranes were clearly visible (**e**); *arrows* show the regions of crystalizing hydroxyapatite, *V* shows the mineralizing vesicle, *white arrowheads* show small vesicles without the hydroxyapatite. In non-osteogenic group, cells have all typical organelles (**b**, **d**), including the nucleus, mitochondria and rough endoplasmic reticulum, the microvesicles released by them were complex but did not contain any electron-heavy crystals (**f**); *N* nucleus, *arrows* alginate fibers, *V* membrane microvesicle; *scale bars* 1 µm (**a**, **b**), 400 nm (**c**, **d**), 200 nm (**e**, **f**)

Block-face scanning electron microscopy of FIB-polished surfaces confirmed that the interior of BMSC from osteogenic group was abundant with electron-opaque structures of crystalline structure. There was no nuclei or organelles visible, and the cell was filled with hydroxyapatite deposits (Fig. 6a). Observations revealed that around the cell there were numerous rounded vesicles containing hydroxyapatite crystals. Near these structures, small vesicles of ~150 nm with low crystal content could be found (Fig. 6c, e). In control cells maintained in non-osteogenic medium, the crystalline structures were absent, and the cellular ultrastructure was normal, with all organelles present within the cytoplasm (Fig. 6b, d). Moreover, in control group the alginate fibers were closer to cell and more visible. Microvesicles released by cell in control group did not contain any crystalline, electron-dense structures (Fig. 6f).

**Fig. 7:**
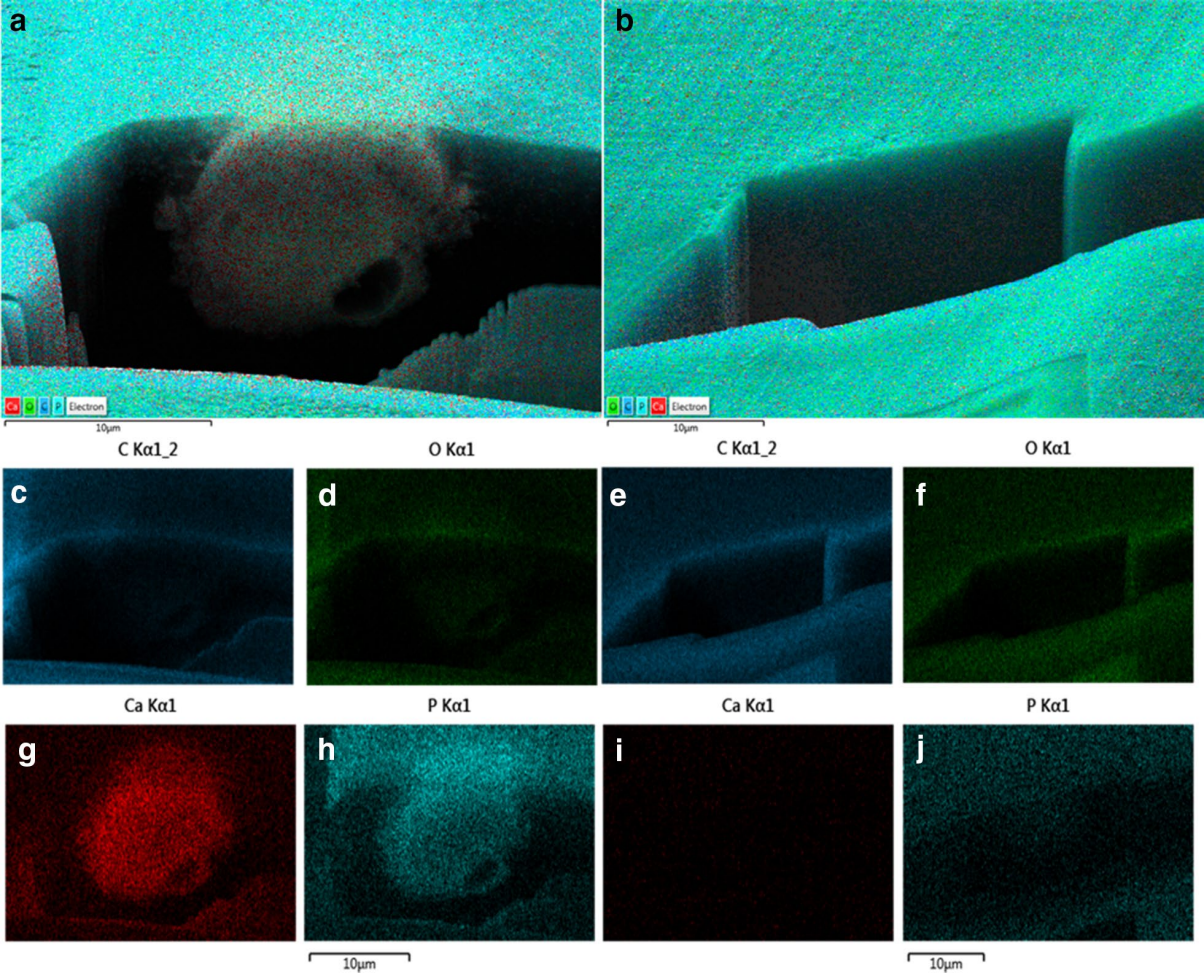
SEM–EDX mapping of calcium and phosphorus concentration in cells from osteogenic (**a**, **c**, **d**, **g**, **h**) and non-osteogenic (**b**, **e**, **f**, **i**, **j**) groups; *multilayered images* indicate the co-localization of specified elements (**a**, **b**), *lower images* indicate the distribution of single elements (carbon **c**, **e**; oxygen **d**, **f**; calcium **g**, **i**; phosphorus **h**, **j**); *scale bars* indicated on micrographs

The SEM–EDX mapping at the FIB-polished surfaces revealed strong signal derived from phosphorus and calcium in osteogenic group, while in non-osteogenic group there was no significant concentration of these elements observed (Fig. 7).

### Measurements of alkaline phosphatase, osteocalcin and osteopontin

**Fig. 8:**
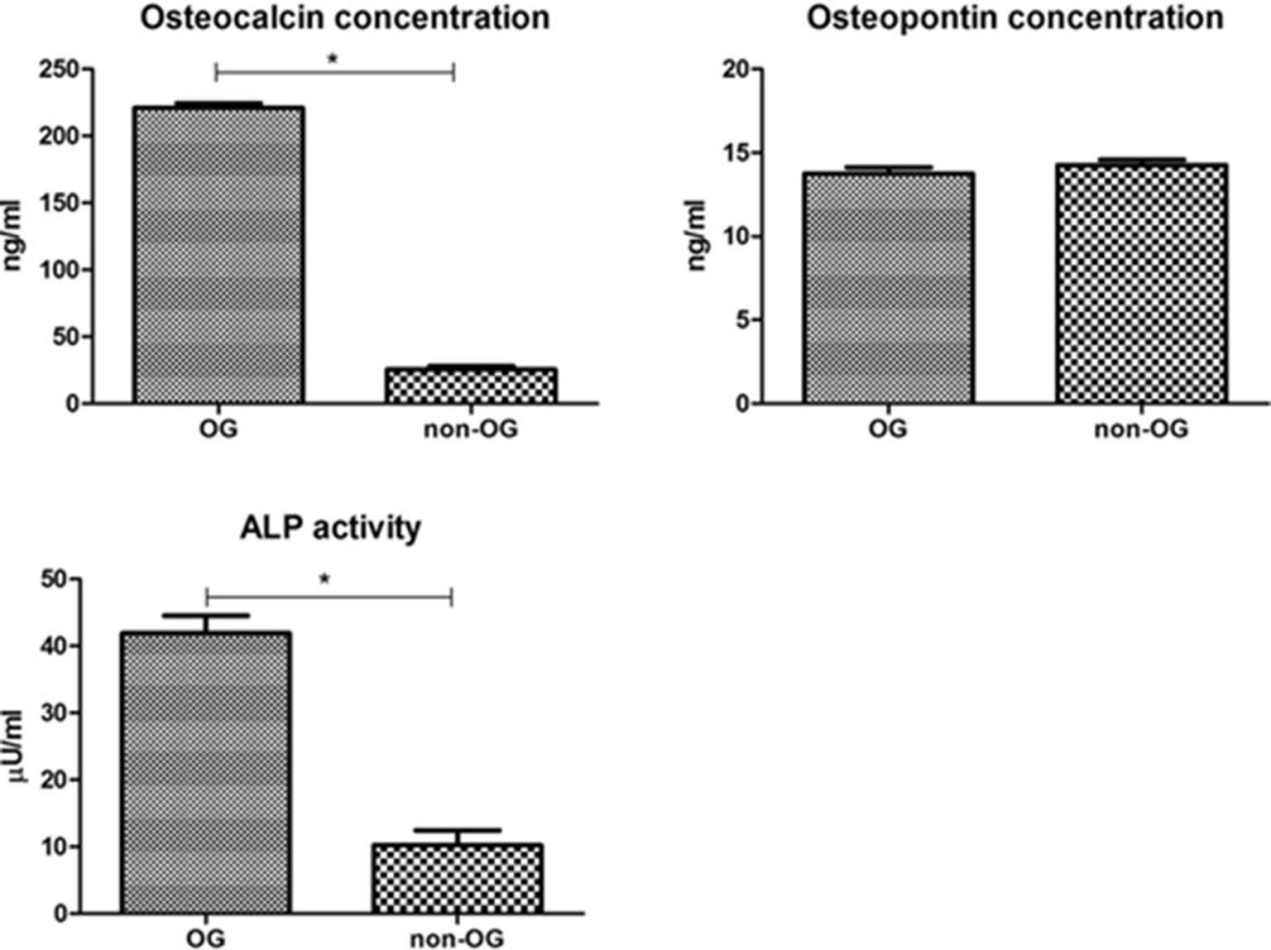
Differences in the activity of alkaline phosphatase, and osteocalcin and osteopontin concentrations in cell culture supernatants after 3 weeks of experiment between osteogenic (OG) and non-osteogenic (non-OG) culture conditions; *p < 0.01

The analysis of alkaline phosphatase activity showed significant differences between experimental and control group (p < 0.01). Samples from osteogenic culture showed the presence of active alkaline phosphatase, while in samples from control cultures its level was 5 times lower. The measurement of osteocalcin concentration in culture supernatants showed significant increase in osteogenic group, in comparison to control, where its level was 10 times lower (p < 0.01). The concentration of osteopontin was comparable in both investigated groups, with no statistically significant difference noticed (p > 0.05, Fig. 8).

## Discussion

The osteogenic differentiation of MSC is a complex process involving the change of gene expression pattern, protein synthesis and the ultrastructure of cells after the process is completed. There are two distinct paths of cell development after its osteogenic differentiation described in the literature. In the first one osteoblast gets on the surface of bone and becomes the bone lining cell after the synthesis of ECM. In the second path osteoblast stays within the ECM and becomes an osteocyte. However, Jilka et al. described, that the number of osteoblasts in the bone growth zone is not equal to the number of cells present in this area after the tissue development is finished, which suggests that some part of cells undergo cellular death [17]. Our results showed that the significant number of cells undergo apoptosis, and this process is probably connected to the bone ECM mineralization process. The apoptotic bodies released from dead cells become the nucleation sites of hydroxyapatite crystallization. Such process occurs not only in bone tissues, but also can be observed for example in the blood vessels where it is connected with the apoptosis of vascular smooth muscle cells [14].

Our results confirm these observations, while the majority of MSC underwent osteogenic differentiation have lost their viability during the process via the apoptosis. Despite the occurrence of cellular death, MSC deposited significant amount of calcium ions within the hydrogel, which was confirmed by the Alizarin Red staining. It should be mentioned however, that in control culture cells also produced and deposited calcium ions, but the histochemical reaction was observed only at the single cell sites, while the majority of hydrogels volume was not stained. This suggest that calcium alginate hydrogel might exert pro-osteogenic effect even without the presence of osteogenic culture medium, which is consistent with observations of other groups, though it is known that particular biomaterials may induce osteogenesis of MSC [18–21]. In the control group the Alizarin Red staining showed no reaction at the hydrogels interior, because the calcium bounded with alginate carbohydrates do not induce the precipitation of a dye. We assume that prominent staining reaction noticed within the superficial zone of hydrogel was caused by its slow dissolution in culture medium and release of calcium ions.

Microscopic observations of BMSC during osteogenic differentiation in alginate hydrogel showed, that the structure of biomaterial did not allow cells for migration and formation of close intercellular contact. Thus in our experiment, cells did not develop multicellular clusters, typical for osteogenic differentiation of cellular monolayer as observed by us and others [1, 22, 23], but were evenly dispersed. We decided to immobilize cells at low concentration to avoid the cell clustering process, so we could analyze the osteogenic differentiation of single cells. As it was reported before, the cell–cell interaction plays significant role during osteogenic differentiation [24]. Our results demonstrate however, that this feature is not crucial for initializing the deposition of bone-like ECM. The Alizarin Red S showed strong reaction of single cells with no developed, multicellular clusters. Therefore, the signals derived only from osteogenic medium, enhanced probably by paracrine cellular communication were sufficient enough for inducing the osteogenic differentiation in mesenchymal stem cells.

Microscopic observations of cellular ultrastructure revealed that BMSC during osteogenic differentiation lost their normal morphology and became filled with hydroxyapatite crystals. Moreover, the alginate fibers were not visible at the cell proximity, which suggest that the calcium composing the forming hydroxyapatite could derive from cross-linking sites of alginate chains, resulting in alginate dispersion. The numerous vesicles of size between 100 and 500 nm and diverse surface morphology were observed at the cell surface and its proximity. This observation corresponds with results obtained by Li et al., where osteoblasts cultured on chitosan/alginate scaffolds were covered with microvesicles containing the calcium-rich minerals [25]. The connection between vesicles and biomineralization has been found by other groups [8–13, 26]. This hypothesis can now be supported by our observations. There were no organelles like nucleus or mitochondria visible within the cell, instead the cell interior was filled with hydroxyapatite crystals of various sizes. We assume that the cellular components become the mineralization sites for growing hydroxyapatite crystals, probably by certain autophagocytic processes. Our observations indicated that the caspase-3 signals were present outside the nuclear area, while the shape of the signal volume might resemble the mineralized microvesicles present around cells. Therefore, it is an another clue connecting the process of apoptosis and biomineralization. As we have shown recently, the macro autophagy is connected to the process of chondrogenic differentiation [27]. Therefore, the similar processes may be involved in the osteogenesis at the cellular level, while these two lineages are relatively similar. In control cells, the three weeks of culture in alginate hydrogel did not alter the proper morphology of cells, leaving numerous mitochondria, endosomes and prominent endoplasmatic reticulum within the cytoplasm. The mineralizing microvesicles were absent on cellular surface, which presented more fibrous structure.

The surface of cells from osteogenic group was covered with round, vesicular objects, with single nanometer-scale fibers. Observations of the inner ultrastructure using ESB detector revealed high heterogeneity in the distribution of areas composed of heavier and lighter elements within the cell. While the calcium and phosphorus have higher mass than carbon, they give higher contrast when observed with backscattered electron detectors. Therefore we concluded that areas of higher contrast seen with ESB detector are in fact the calcium and phosphorus deposits, which was confirmed by SEM–EDX mapping. These observations supports the hypothesis that during the osteogenic differentiation, matrix vesicles accumulate the calcium within their interior [13]. Though in non-osteogenic group the vesicular structures inside cells were numerous, they did not show the presence of heavier elements inside. Observations performed with block-face low voltage imaging technique revealed the presence of expanded apatite-like structures of irregular shape concentrating in the round, membrane vesicles, supporting the theory of mineralizing role of these structures.

In non-osteogenic group the majority of cells were alive and of normal morphology and ultrastructure. Despite the lack of adhesion sites in the pure calcium alginate, cells survived three weeks of culture and did not change their appearance. The mineralized microvesicles were absent in cells from control group, and the alginate fibers were tightly packed around the cell.

The effectiveness of osteogenic stimulation was also reflected by the increased concentrations of osteocalcin secreted to the supernatants and the activity of ALP. As it should be expected, the quantity of osteopontin in media was comparable in both groups. Osteopontin, beside the role in osteogenesis, is also considered as important factor in cell proliferation, and it is actively synthesized also in not differentiated MSC [28, 29]. Analysis of calcium and phosphorus content using EDX showed higher concentrations of both elements in hydrogels from osteogenic conditions. Moreover, the calcium-to-phosphorus ratio in experimental group was almost exact 2-to-1, like in natural bone [30]. All observations confirmed the positively induced osteogenic differentiation in experimental group.

## Conclusions

The mechanisms responsible for production and deposition of ECM in the 3D-culture in vitro model using mesenchymal stromal cells seems to be similar to natural osteogenesis occurring in vivo. We showed at the ultrastructural level that the deposition of mineralized extracellular matrix during osteogenic differentiation is connected with cell dead, where cellular remnants become the nucleation sites of mineralized matrix. This process probably involves the cell apoptotis and autophagocytosis, however the exact physiological mechanisms directing the cell death still need to be investigated.

## Additional file

**Additional file 1.** Movie caption of 3D-reconstruction of cell exhibiting the presence of caspase-3, showing the extranuclear localization of the immunofluorescence reaction.

## Abbreviations

MSC: mesenchymal stem cells
MV: microvesicles
HBSS: Hank’s balanced salt solution
DMEM: Dulbecco’s modified eagle medium
BMSC: bone marrow-derived mesenchymal stem cells
MACS: magnetic-activated cell sorting
CPD: critical point drying
UPW: ultrapure water
TCH: thiocarbohydrazide
FIB: focused ion beam
HM: heavy metal
EDX: energy dispersive X-ray spectrometry
ALP: alkaline phosphatase
OCL: osteocalcin
OPN: osteopontin
